# Topological Nodal Surface and Quadratic Dirac Semimetal States and van Hove Singularities in ScH_3_ and LuH_3_ Superconductors

**DOI:** 10.1021/acsomega.3c00207

**Published:** 2023-03-01

**Authors:** Ali Sufyan, J. Andreas Larsson

**Affiliations:** Applied Physics, Division of Materials Science, Department of Engineering Sciences and Mathematics, Luleå University of Technology, Luleå SE-97187, Sweden

## Abstract

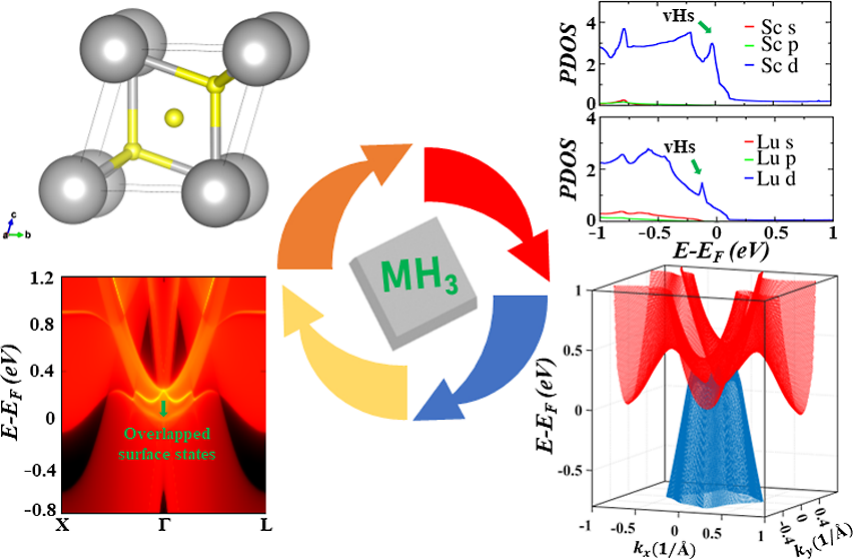

The coexistence of non-trivial topology and superconductivity in a material may induce a novel physical phenomenon known as topological superconductivity. Topological superconductors have been the subject of intense research, yet there are severe limitations in their application due to a lack of suitable materials. Topological nodal surface semimetals with nearly flat nodal surfaces near the Fermi level can be promising materials to achieve topological superconductivity. Here, we use first-principles calculations to examine the topological electronic characteristics of two new superconductors, ScH_3_ and LuH_3_, at both ambient and high pressures. Our studies show that both ScH_3_ and LuH_3_ have van Hove singularities, which confirms their superconductivity. Interestingly, both materials host topological nodal surface states under the protection of time reversal and spatial inversion symmetries in the absence of spin–orbit coupling (SOC). These nodal surfaces are distinguished by a pair of unique drum-head-like surface states not previously observed in nodal surface semimetals. Moreover, the nodal surfaces transform into essential spin–orbit quadratic Dirac points when SOC is included. Our findings demonstrate that ScH_3_ and LuH_3_ are good candidates to investigate the exotic properties of both nodal surface semimetals (NSSMs) and quadratic Dirac semimetal states and also provide a platform to explore the coexistence of topology and superconductivity in NSSMs with promising applications in high-speed electronics and topological quantum computing.

## Introduction

During the past decade, topological states of matter have emerged as a major area of research in condensed matter physics.^1−3^ With inspiration from earlier work on topological insulators,^4−16^ the research focus is now shifting toward gapless topological phases, particularly topological semimetals (TSMs).^17−22^ TSMs exhibit nontrivial band crossings (BCs) between valence and conduction bands in momentum space protected by certain symmetries, such that the quasiparticles behave drastically different from the conventional Schrödinger-type fermions. For instance, in Dirac^23−26^ and Weyl semimetals,^27−30^ the zero-dimension (0D) BCs between conduction and valence bands, accompanied by fourfold and twofold degeneracies, occur at isolated k points around which the low-energy quasiparticles resemble relativistic Dirac and Weyl fermions, allowing high-energy physics to be simulated in a desktop setting. In addition to Weyl/Dirac SMs, nodal line semimetals^31−39^ (NLSMs) form another class of SMs that host 1D BCs and have also been extensively pursued over the past few years. Recently, a new class of SMs referred to as nodal surface semimetals^40,41^ (NSSMs) has also been proposed, initially by Zhong et al.^42^ and Liang et al.^43^ in carbon networks and BaVS_3_ family materials, respectively.

NSSMs possess 2D BCs, where each point on the surface is a crossing point between conduction and valence bands with linear dispersion along the surface normal direction.^40^ Wu et al.^40^ divide NSSMs into two categories based on symmetry protection mechanisms and also provide sufficient conditions and examples for robust NSSMs under spin–orbit coupling (SOC) and in magnetic materials. However, the proposed NSSMs are far fewer than Weyl-Dirac and NLSMs, and the number is even smaller when it comes to their experimental realization.^44−48^ NSSMs are anticipated to hold interesting topological physics and many promising applications in devices. To study these properties from a material point of view, it is crucial that the band degeneracies are close to the Fermi level. Moreover, for experimental realization, the material should be stable and easy to synthesize. These rigorous conditions limit the development of suitable NSSMs, and there is an urgent need to search for realistic materials that are suitable for the experimental study of NSSM states.

In parallel, topological superconductors have attracted much attention in recent years because they can host Majorana zero modes and have potential applications in topological quantum computation.^49−53^ Despite great efforts to develop topological superconductors, here a severe shortage of suitable materials has hindered their development.^50^ Experimentally, doping a topological material to turn it into a superconductor or tuning a superconducting material into a topological phase via doping are common methods to realize new topological superconductors.^54−57^ In addition, topological superconductivity can also be achieved by topological materials that can be driven into a superconducting phase by pressure.^58,59^ However, the ideal approach to realize new topological superconductors is to find a single material that possesses both topological and superconducting properties simultaneously.^60−66^

Polyhydrides are a large class of materials that have undergone extensive research in a variety of fields, including energy storage^67^ and superconductivity.^68−72^ The chemical precompression of polyhydrides makes them an excellent candidate for high-temperature superconductors at feasible pressures.^73^ Rare-earth metal hydrides LaH_10_ and YH_10_ are predicted to be surprising high-temperature superconductors at megabar pressures,^74−78^ and several rare-earth metal hydrides, including CeH_9_,^79^ CeH_10_,^79^ YH_6_,^80^ and YH_9_,^81^ have been experimentally demonstrated to be superconductors with promisingly high *T*_c_. Recently, two new hydrides, ScH_3_ and LuH_3_, have also been experimentally synthesized and found to exhibit superconductivity, with *T*_c_ ∼ 18.5 K at 131 GPa and 12.4 K at 122 GPa,^82^ respectively. ScH_3_ and LuH_3_ are the only REH_3_ hydrides whose superconducting properties have been experimentally confirmed. Despite the fact that both of these hydrides show promise for superconductivity, research on their electronic topological properties has yet to be done.

For the aforementioned reasons, we have examined the topological features of superconducting hydrides, ScH_3_ and LuH_3_^82^ at both ambient and high pressures in the present study. Our first-principles calculations show that these materials exhibit U-shaped nodal surface states in the absence of SOC. When SOC is included, the nodal surface states split and transform into an essential quadratic Dirac cone^83^ that is located very close to the Fermi level. Furthermore, we also identified the presence of van Hove singularities (vHss) in ScH_3_ and LuH_3_, which verifies the superconductive nature of these materials. Thus, our work provides a promising material platform for exploring the intriguing properties of the nodal surface and quadratic Dirac semimetal states, as well as new candidates exhibiting topological and superconducting properties simultaneously.

## Computational Details

First-principles calculations based on density functional theory^84^ were performed using the projector augmented wave^85^ method as implemented in the Vienna ab initio simulation (VASP)^86,87^ package. The exchange–correlation functional was treated using the Perdew–Burke–Ernzerhof generalized gradient approximation (GGA)^88^ with the cutoff energy set to 450 eV. The Brillouin-zone (BZ) integration was sampled using 18 × 18 × 18 Γ-centered Monkhorst–Pack grids.^89^ The structures were relaxed until the residual force on each atom was less than 10^–3^ eV/Å. The energy convergence criterion for electronic structure calculations was set to 10^–6^ eV. The SCAN^90^ meta-GGA energy functional was used to correct the underestimated band gaps under GGA. The raw output files of VASP were extracted and analyzed using the VASPKIT code.^91^ The real-space tight-binding model Hamiltonians were constructed by using the VASP2WANNIER90 interface,^92^ and Sc/Lu d and H s states were included in generating Wannier functions. The surface electronic structures were calculated using the WannierTools package.^93^

## Results and Discussion

**Figure 1:**
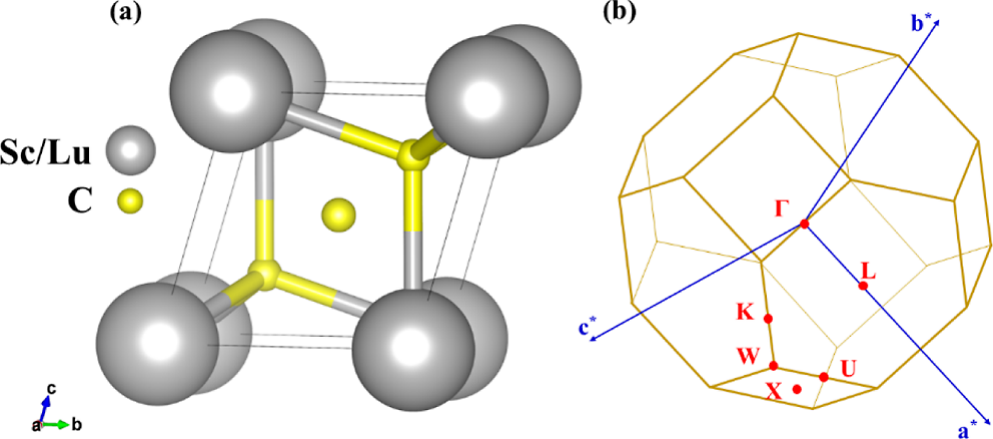
(a) Side view of the crystal structure. The gray and yellow spheres represent the Sc/Lu and H atoms, respectively. (b) Corresponding bulk BZ.

Both scandium and lutetium tri-hydrides crystallize in a cubic configuration with the space group *Fm*3_*m* (no. 225). The primitive unit cell of these trihydrides includes one Sc/La atom and three H atoms, as shown in Figure 1a. The Sc/Lu atom is located at (0, 0, 0), while the H atoms are located at (0.25, 0.25, 0.25), (0.75, 0.75, 0.75), and (0.5, 0.5, 0.5) positions. Since it has been reported that these compounds exhibit superconductivity at 131 and 122 GPa,^82^ respectively, their electronic and topological properties have been simulated at both ambient and high pressures (131 GPa for ScH_3_ and 122 GPa for LuH_3_). Both compounds display similar electronic and topological properties at ambient and high pressure; the ambient-pressure results will be discussed here, whereas the high-pressure results are given in the Supporting Information. The calculated lattice parameters at ambient pressure for ScH_3_ and LuH_3_ are *a* = *b* = *c* = 3.36 Å and 3.53 Å, respectively, and the cell angles are α = β = γ = 60°. Furthermore, the crystal structure possesses inversion symmetry and also respects time-reversal symmetry. Figure 1b displays the corresponding BZ with high-symmetry points.

**Figure 2:**
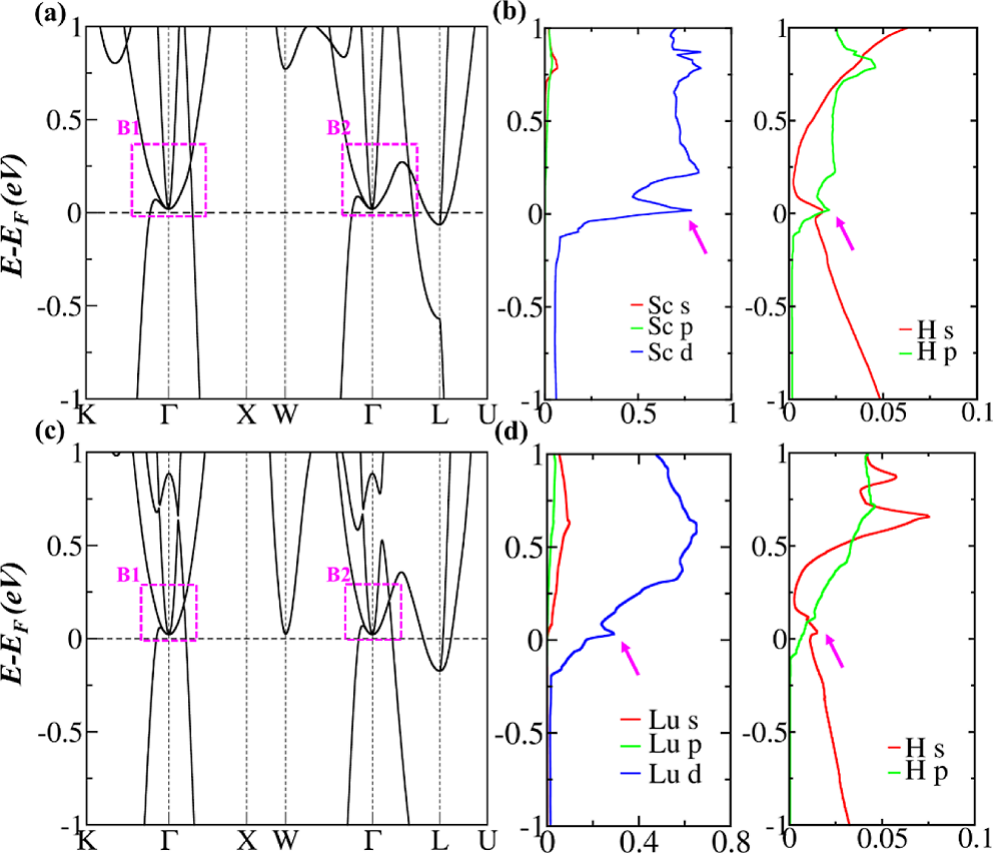
Electronic band structures and orbital projected DOS for (a,b) ScH_3_ and (c,d) LuH_3_ using the SCAN functional. The high-symmetry *k* points are given in Figure 1b. The magenta arrows in (b,d) point to the diverging DOS.

The calculated SCAN electronic band structures for ScH_3_ and LuH_3_ are given in Figure 2a,c, respectively, in the absence of SOC. In both materials, the bands are mostly confined to two regions labeled as B1 and B2 and share similar semimetallic characteristics. Near the Fermi level, the conduction band maximum (CBM) and valence band minimum (VBM) exactly stick together in both regions and form unique U-shaped nodal surfaces while they are fully gapped beside these regions. Figure 2b,d presents the element-projected density of states (DOS) for ScH_3_ and LuH_3_, respectively. It is evident that the bands near the Fermi level are mainly contributed by the d orbitals of Sc/Lu atoms that are hybridized with the s orbitals of H atoms (the Lu f orbitals make no contribution to the bands near the Fermi level). The hybridization strength is the strongest in ScH_3_ as it has a small lattice constant,^94,95^ which causes flatness of the degenerate bands around Γ, resulting in a wider U-shape of the degenerate bands in ScH_3_ (see Figure 2a,c). With flat bands, many allowed states occupy almost the same energy levels, resulting in a high or diverging DOS, a characteristic feature of vHs.^96−98^ It is evident that the vHs is present in the DOS of ScH_3_ and LuH_3_ (indicated by the magenta arrows in Figure 2b,d), demonstrating that superconductivity emerges in these materials due to the presence of flat bands near the Fermi level.

**Figure 3:**
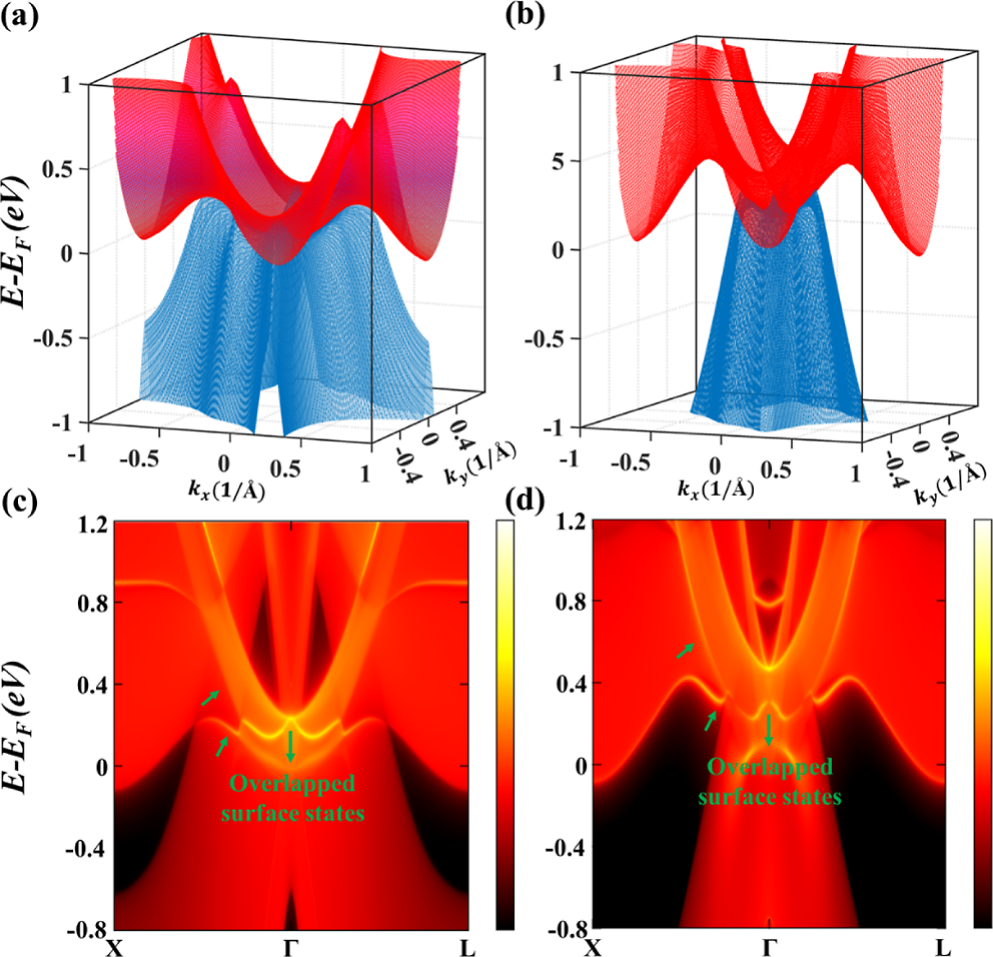
3D band structures under SCAN without SOC for (a) ScH_3_ and (b) LuH_3_ near the nodal lines form in the vicinity of the Fermi level. The calculated (100) surface band structures for (c) ScH_3_ and (d) LuH_3_ along the projected *X*–Γ–*Z k*-path without SOC.

To confirm the shape of topological nodal surfaces, the 3D energy bands with Γ-point at the middle for ScH_3_ and LuH_3_ are computed without SOC in Figure 3a,b, respectively. There is a clear U-shaped overlap between VBM and CBM around Γ, which corresponds to the nodal surfaces formed due to the band touching points in ScH_3_ and LuH_3_. Besides, an important characteristic of topological materials is their novel surface states. TIs exhibit helical surface states, while materials with topological nodal lines usually exhibit drumhead-like surfaces without SOC. However, the surface states of NSSMs are not evident since, to the best of our knowledge, they have never been reported. Figure 3c,d displays the calculated surface energy spectra for ScH_3_ and LuH_3_, respectively, in the absence of SOC. Interestingly, the surface spectra of both NSSM materials contain two drumhead-like surface states pointed by green arrows which are entirely overlapped around Γ and get separated as we move away from Γ in both directions. These findings provide an insight into how NSSM’s surface states can appear, which can be used to identify their nodal surface states in experiments.

**Figure 4:**
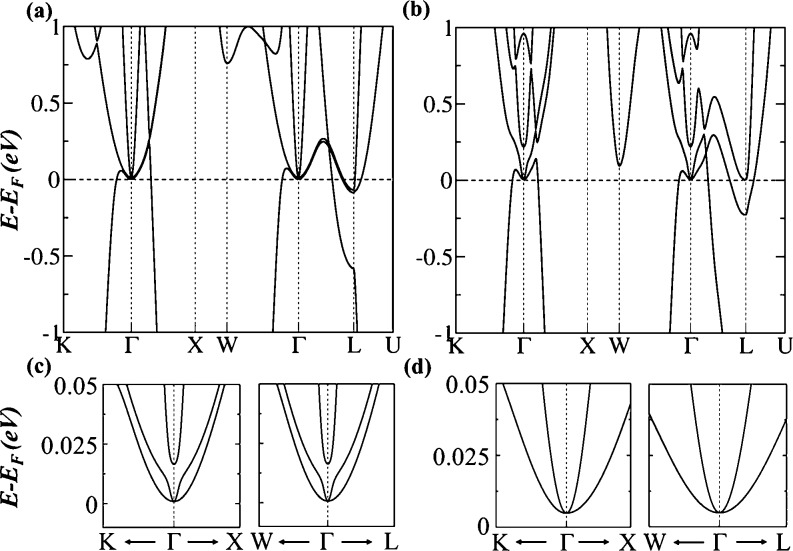
Calculated electronic band structures with SOC for (a) ScH_3_ and (b) LuH_3_ using the SCAN functional. Panels (c,d) are the zoomed-in images for the low-energy bands along *K*–Γ–*X* and *W*–Γ–*L*, showing two Dirac points at Γ for both ScH_3_ and LuH_3_, respectively.

Next, we examine how SOC affects the electronic and topological properties of ScH_3_ and LuH_3_. Figure 4 shows the electronic band structures of ScH_3_ and LuH_3_ with SOC. Clearly, the 2D nodal surfaces have been removed, and a small gap between VBM and CBM has appeared along *K*–Γ, Γ–*X* and *W*–Γ, Γ–*L*, as shown in zoomed-in plots in Figure 4c,d. However, in both materials, the VBM and CBM remain connected at Γ and form Dirac points. Furthermore, the dispersion of these Dirac points is quadratic^83^ rather than linear, and they are robust against SOC. In fact, these Dirac points only appear in the presence of SOC.

**Figure 5:**
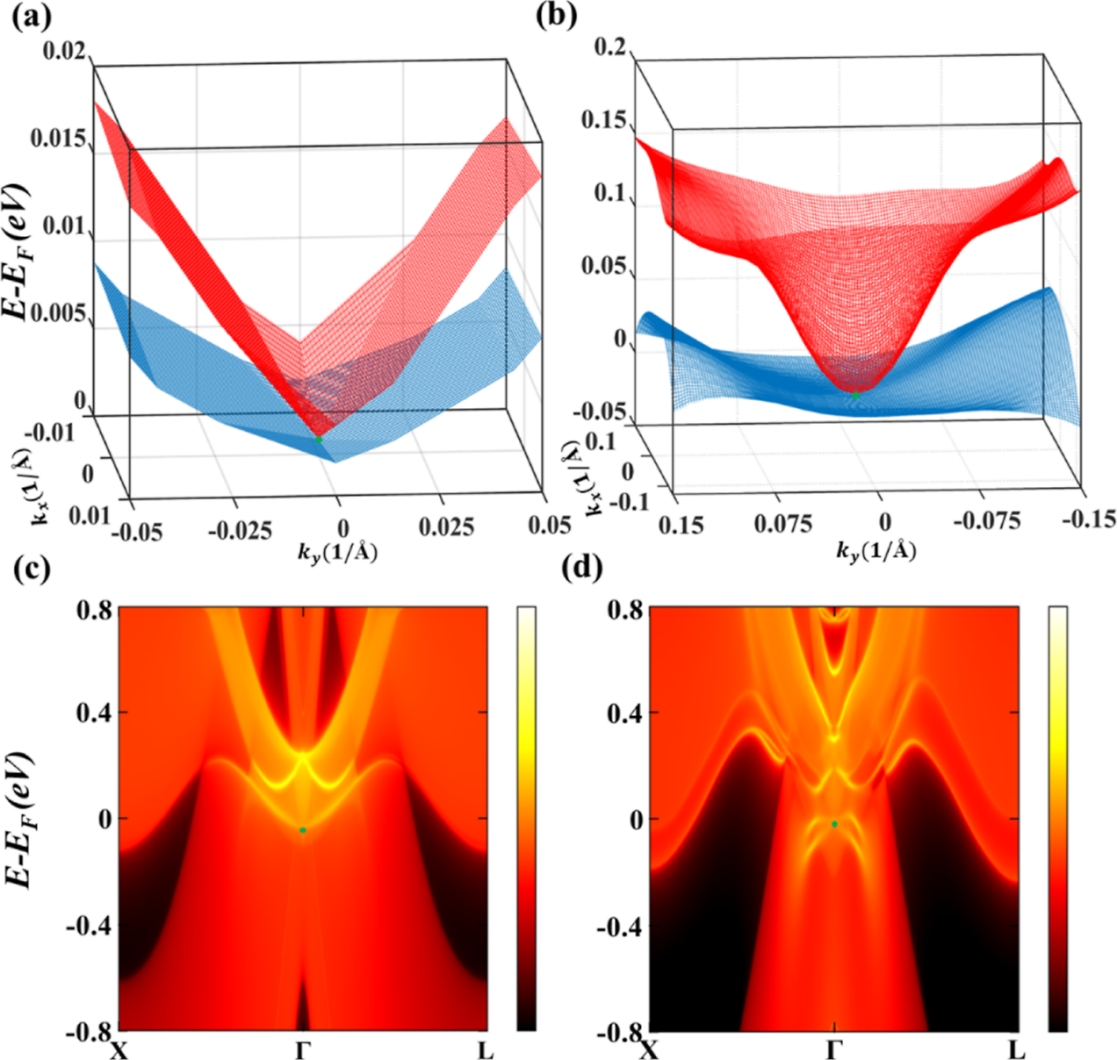
3D band structures under SCAN with SOC for (a) ScH_3_ and (b) LuH_3_ near the Dirac points form in the vicinity of the Fermi level. (c) Surface band structures calculated along the projected *X*–Γ–*Z k*-path for ScH_3_ and (d) LuH_3_ with SOC. The position of bulk Dirac points is denoted by the green points.

To support the findings, 3D energy bands of ScH_3_ and LuH_3_ are depicted in Figure 5a,b, respectively, demonstrating a clear crossing between the VBM and CBM at Γ. Following that, the surface states of ScH_3_ and LuH_3_ are investigated under SOC (see Figure 5c,d). The two drumhead surface states, which were previously overlapped in the absence of SOC, are now completely gapped, as expected in type-I NSSMs.^40^ Besides, the bulk states are mixed with each other around Γ, and the locations of bulk Dirac cones could be faintly visible as indicated by the green dots.

Our simulations show both superconducting and topological properties for ScH_3_ and LuH_3_. Since these two materials have been realized experimentally, we suggest they be used as a platform to explore this combination of properties for applications in spintronics and topological quantum computation. Our theoretical findings suggest that the NSSM states and their unique properties are more likely to be observed in ScH_3_ since the SOC-induced gap is extremely small around its nodal surfaces.

## Conclusions

In summary, on the basis of first-principles calculations, we predicted the existence of U-shaped nodal surface states in two superconductors, ScH_3_ and LuH_3_, in the absence of SOC. The nodal surfaces are formed by a continuous overlap of VBM and CBM around Γ and exhibit drum-head-like surface states that have not been reported before. With the inclusion of SOC, the nodal surfaces weakly split and transform into quadratic Dirac points. Besides, both materials contain vHss, proving their superconductive nature. Considering the fact that the SOC-induced gaps around the nodal surfaces are small, especially in the ScH_3_ case, these materials are promising to serve as good candidates to study both NSSMs and quadratic Dirac semimetal states and also provide a platform to explore the coexistence of topology and superconductivity in NSSMs.

## Supporting Information Available

Electronic band structures and partial density of states of ScH_3_ and LuH_3_ without SOC at 140 and 122 GPa pressure, respectively; 3D band structures and surface states of ScH_3_ and LuH_3_ at 140 and 122 GPa, respectively, without SOC; electronic band structures of ScH3 at 140 GPa and LuH_3_ at 122 GPa with SOC; 3D band structures and surface states for ScH_3_ at 140 GPa and LuH_3_ at 122 GPa with SOC; and the zoomed-in view of 3D band structures of ScH_3_ and LuH_3_ at ambient pressure without SOC near the Dirac points (PDF)

## Notes

The data that support the findings of this study are available from the corresponding author upon reasonable request.
