# Supplementary material for: Topological Nodal Surface and Quadratic Dirac Semimetal States and van Hove Singularities in ScH3 and LuH3 Superconductors
Source: ACS Omega. 2023 Mar 1;8(10):9607–13. doi: 10.1021/acsomega.3c00207 (PMC10018709; doi:10.1021/acsomega.3c00207)
Supplement: Supplementary file 1 — ao3c00207_si_001.pdf [file ao3c00207_si_001.pdf]

Supplementary Information

Topological nodal surface and quadratic Dirac semimetal states and van Hove singularities in ScH_3 and LuH_3 superconductors

Ali Sufyan*, J. Andreas Larsson

Applied Physics, Division of Materials Science, Department of Engineering Sciences and Mathematics, Luleå University of Technology, Luleå SE-97187, Sweden

*Corresponding author E-mail: ali.sufyan@associated.ltu.se Tel: +46 761368932

Figure S1 displays the electronic band structures and partial density of states of ScH_3 and LuH_3 without spin-orbit coupling (SOC) at 140 GPa and 122 GPa pressure, respectively. Figure S2 shows the 3D band structures and surface states of ScH_3 and LuH_3 at 140 GPa and 122 GPa, respectively, without SOC. Figure S3 displays the electronic band structures of ScH_3 at 140 GPa and LuH_3 at 122 GPa with SOC while Figure S4 shows the 3D band structures and surface states for ScH_3 at 140 GPa and LuH_3 at 122 GPa with SOC. Figure S5 displays the zoom-in view of 3D band structures of ScH_3 and LuH_3 at ambient pressure without SOC near the Dirac points.

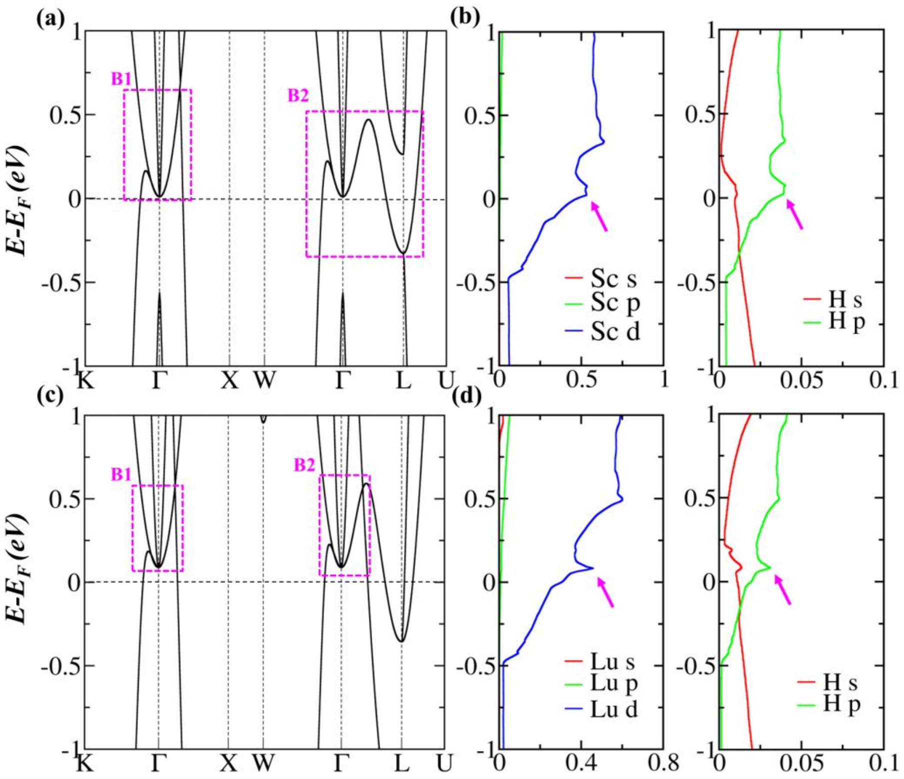

Figure S1. Electronic band structures and orbital projected density-of-states (DOS) for (a, b) ScH_3 at 140 GPa and (c, d) LuH_3 at 122 GPa without SOC using SCAN functional. The magenta arrows in (b) and (d) point to the diverging DOS.

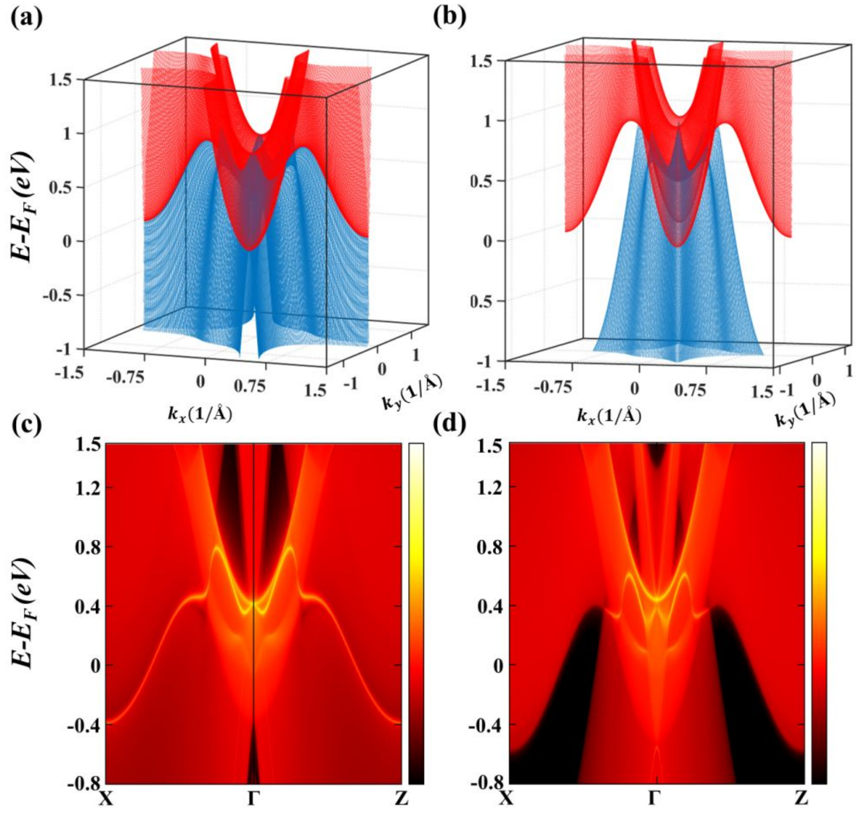

Figure S2. The 3D band structures under SCAN without SOC for (a) ScH_3 at 140 GPa and (b) LuH_3 at 122 GPa near the nodal lines form in the vicinity of Fermi level. The calculated (100) surface band structures for (c) ScH_3 and (d) LuH_3 along the projected X–Γ–Z k-path without SOC.

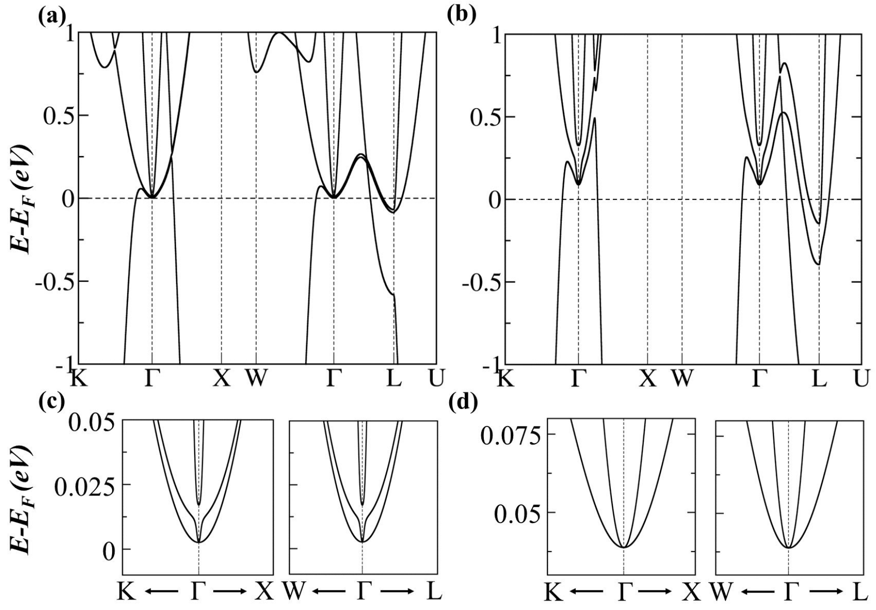

Figure S3. Calculated electronic band structures with SOC for (a) ScH_3 at 140 GPa and (b) LuH_3 at 122 GPa using SCAN functional. Panels (c) and (d) are the zoom-in images for the low-energy bands along K-Γ-X and W- Γ-L showing two Dirac points at Γ for both ScH_3 and LuH_3, respectively.

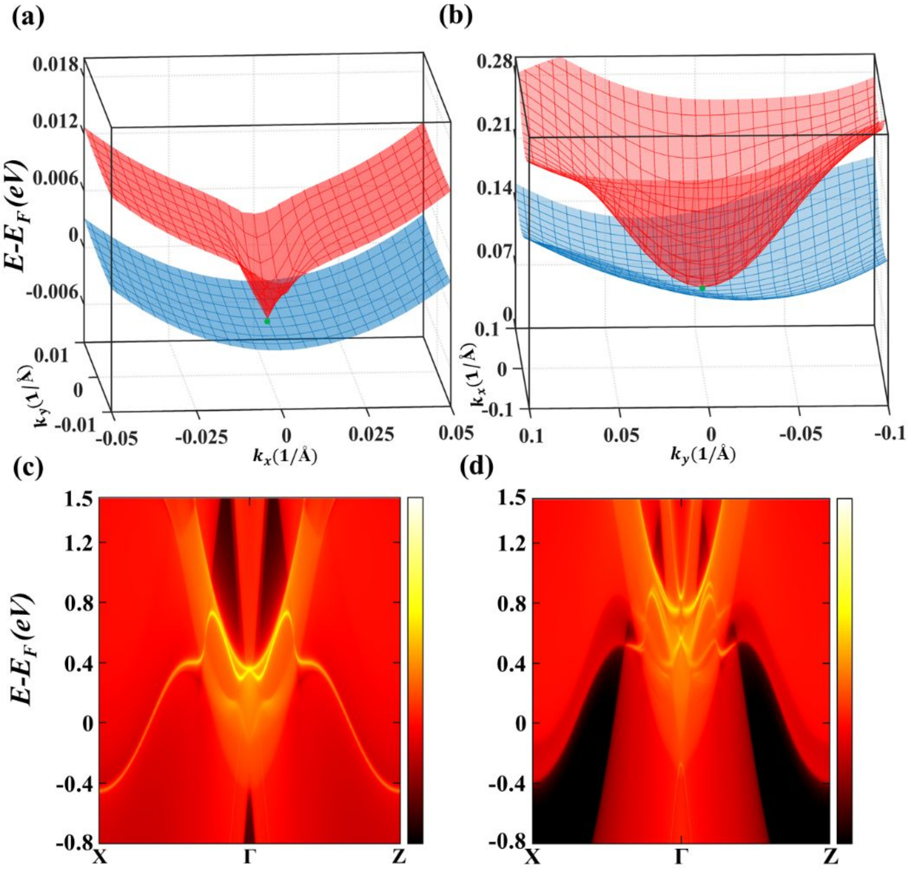

Figure S4. The 3D band structures under SCAN with SOC for (a) ScH_3 at 140 GPa and (b) LuH_3 at 122 GPa near the nodal lines form in the vicinity of Fermi level. The calculated (100) surface band structures for (c) ScH_3 and (d) LuH_3 along the projected X–Γ–Z k-path with SOC.

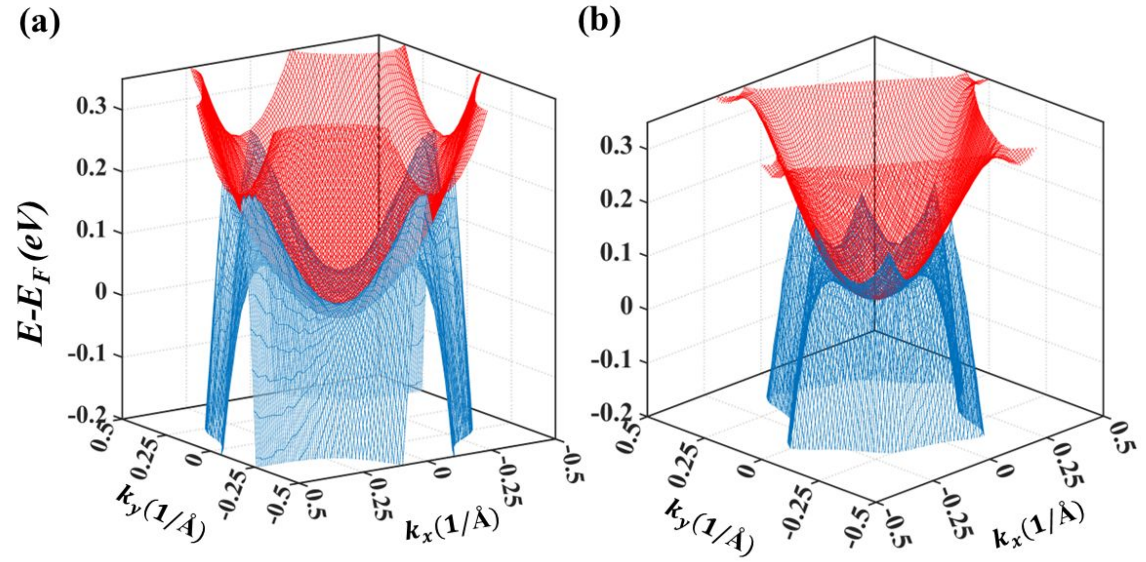

Figure S5. The zoom-in view of 3D band structures under SCAN at ambient pressure without SOC for (a) ScH_3 and (b) LuH_3 near the Dirac points form in the vicinity of the Fermi level.
